# Giving Your Cell Phone Number to Patients

**DOI:** 10.5811/westjem.2016.4.30359

**Published:** 2016-06-13

**Authors:** C. Ferrell Varner

**Affiliations:** University of Tennessee Medical Units at Memphis, Department of Emergency Medicine, Memphis, Tennessee

Below is a letter concerning contact with patients. I have found this practice useful. It decreases anxiety on the part of the patient and the doctor. I write to recommend it to emergency physicians everywhere.

I give my cell phone number to patients all the time. By that I mean 2–3 times a shift. I have been doing it for years, almost since I first got a cell phone. I have given it out hundreds of times. I recommend that we encourage our emergency medicine (EM) residents to do so also. It is an easy option, and it can help avoid all sorts of problems. Discretion is in order, but there are not a lot of exceptions. There are some types of patients that I do not give it to.

I give it to patients for several different reasons. Often I want to know what happened to the patient. Did they get better? Did my recommended treatment work? For instance, they had abdominal pain, and I want to know if they got over it. I say, “Call me in 48 hours and let me know what happened.” Out of 100 of these types of requests I may get one call back. I assume that they get better and that they forget about it. Our medical system is good, and we generally get it right.

I give it to the unsatisfied patient very often. I think you know the interaction. You have finished your workup, and you are discharging the patient. You are giving your summary, and the patient or family member obviously thinks that you have either not done enough, ought to admit the patient, or that you are wrong. They will not state this, but it is obvious that they are not entirely happy. At that point, I say my standard discharge spiel. “With this treatment you should get better and better. If you get worse, notify your doctor immediately. If unable to reach him/her, return immediately to the emergency department (ED). By the way, here is my phone number. Call me if you have a problem or are getting worse. I don’t sleep with the phone, so I may not answer in the middle of the night. If you call once and I don’t answer, call me again. Sometimes I cannot be reached, so if you are worsening and cannot reach me right away, go to the ED immediately. But call me if you have a problem.”

At that point everything changes. The patients usually are very pleased. You have told them two things very clearly. First, you think that your diagnosis and treatment are correct. Second, that you care about them. You are not going to hide behind a wall of secretaries who will not connect you. You believe in your care.

I also give my number to patients that I am a little worried about. I think that I am right. However, I want to make sure that they get timely care if things turn worse. A patient has a tender area that I think is cellulitis, and I treat with an antibiotic. If they get worse, call me.

And what if they do call? It is a 30-second conversation. “Go to the ED. I will call them and tell them to be looking for you.”

During the eight or so years that I have been doing this, I have been called back perhaps six times. It has never been “abused.” Only once did I have to have the patient return to the ED, and it was not a major issue. My patient satisfaction scores are the best in the department.

Physicians in other specialties who see patients repeatedly might have difficulty with this. Because we in the ED do not have ongoing relationships with our patients, this practice should not create a problem. Residents might want to use their beeper numbers instead, until they are comfortable with the process.

